# Supplementary figures and images for: Systems genetics uncover new loci containing functional gene candidates in Mycobacterium tuberculosis-infected Diversity Outbred mice
Source: PLoS Pathog. 2024 Jun 11;20(6):e1011915. doi: 10.1371/journal.ppat.1011915 (PMC11195971; doi:10.1371/journal.ppat.1011915)

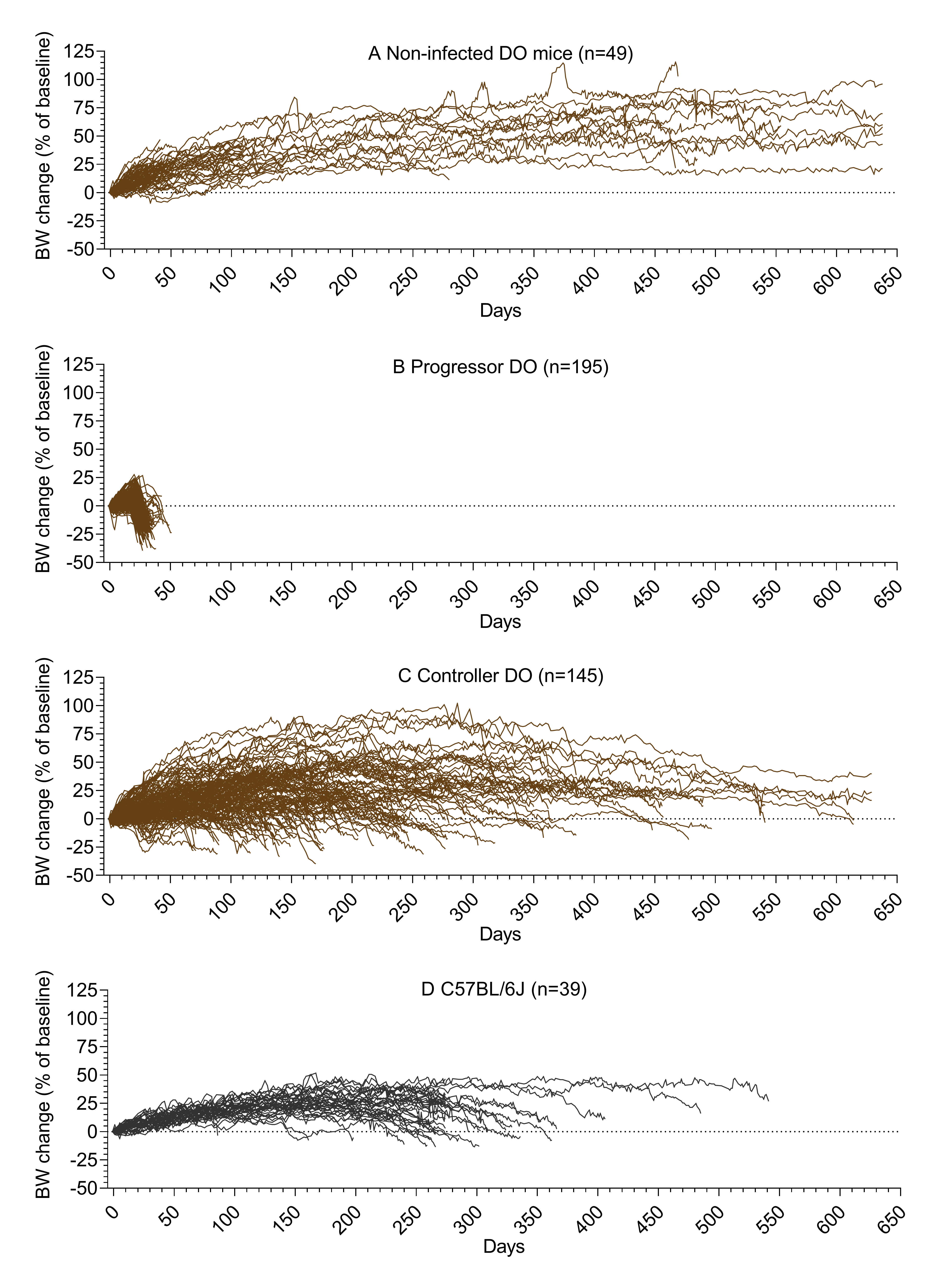

Supplement: S1 Fig — Mice were infected with a low dose of M. tuberculosis strain Erdman by aerosol and infection progressed unmanipulated until mice were euthanized due to IACUC-approved morbidity criteria. Panel A: Body weight changes of identically housed, age-, gender-, and generation-matched non-infected Diversity Outbred (DO) mice (n = 49) compared to baseline. Panels B, C, and D: Body weight changes of Progressor DO mice (n = 195); Controller DO mice (n = 145); and C57BL/6J inbred founder strain mice that succumbed to pulmonary TB (n = 39), are shown over time compared to pre-infection baseline. All mice were weighed 1 to 3 days prior to M. tuberculosis infection, at least twice per week during infection, and immediately before euthanasia. Each line is the body weight expressed as a percent change compared to initial pre-infection body weight. (TIF) [file ppat.1011915.s001.tif]

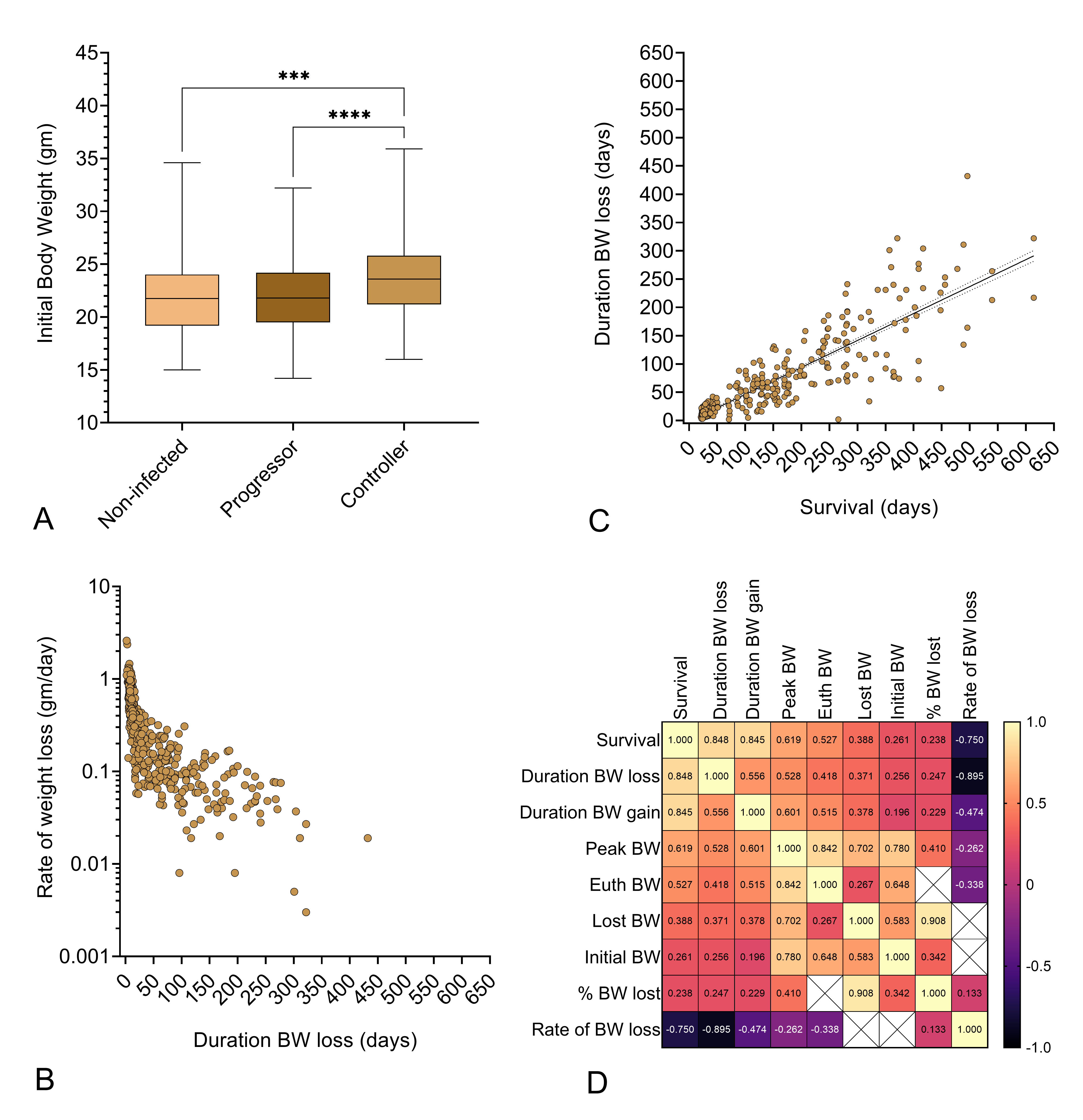

Supplement: S2 Fig — Age-, gender-, and generation-matched DO mice were assigned to cages at random, and infected (or not infected) with a low dose of M. tuberculosis strain Erdman by aerosol exposure. All mice were initially weighed 1–3 days prior to infection, at least twice per week during infection, and immediately before euthanasia. Panel A: shows retrospective analysis of pre-infection body weights of Non-infected mice (n = 76) compared to pre-infection body weights of Progressors (n = 298) and pre-infection body weights of Controllers (n = 195), shown as box-and-whisker plots with the line at the mean for each group, and whiskers at the minimum and maximum. Data were analyzed by 1-way ANOVA with Tukey’s multiple comparisons test ***p<0.001; ****p<0.0001. Panels B, C, D: Infection progressed unmanipulated until mice were euthanized due to IACUC-approved morbidity criteria. Panel B: The rate of weight loss (gm/day) and duration of body weight (BW) loss in days negatively correlate with each other. Panel C: Duration of BW loss was strongly, positively, and linearly correlated with survival by Spearman correlation analysis (r = 0.848 with dashed lines indicating the 95% confidence interval, 0.8204 to 0.8717, p<0.0001). Panel D: Correlation matrix to show how survival and eight clinical indicators correlate with each other. Only correlations with p-values <0.00001 are shown on the matrix. Cells marked by an “X” were not significantly correlated. (TIF) [file ppat.1011915.s002.tif]

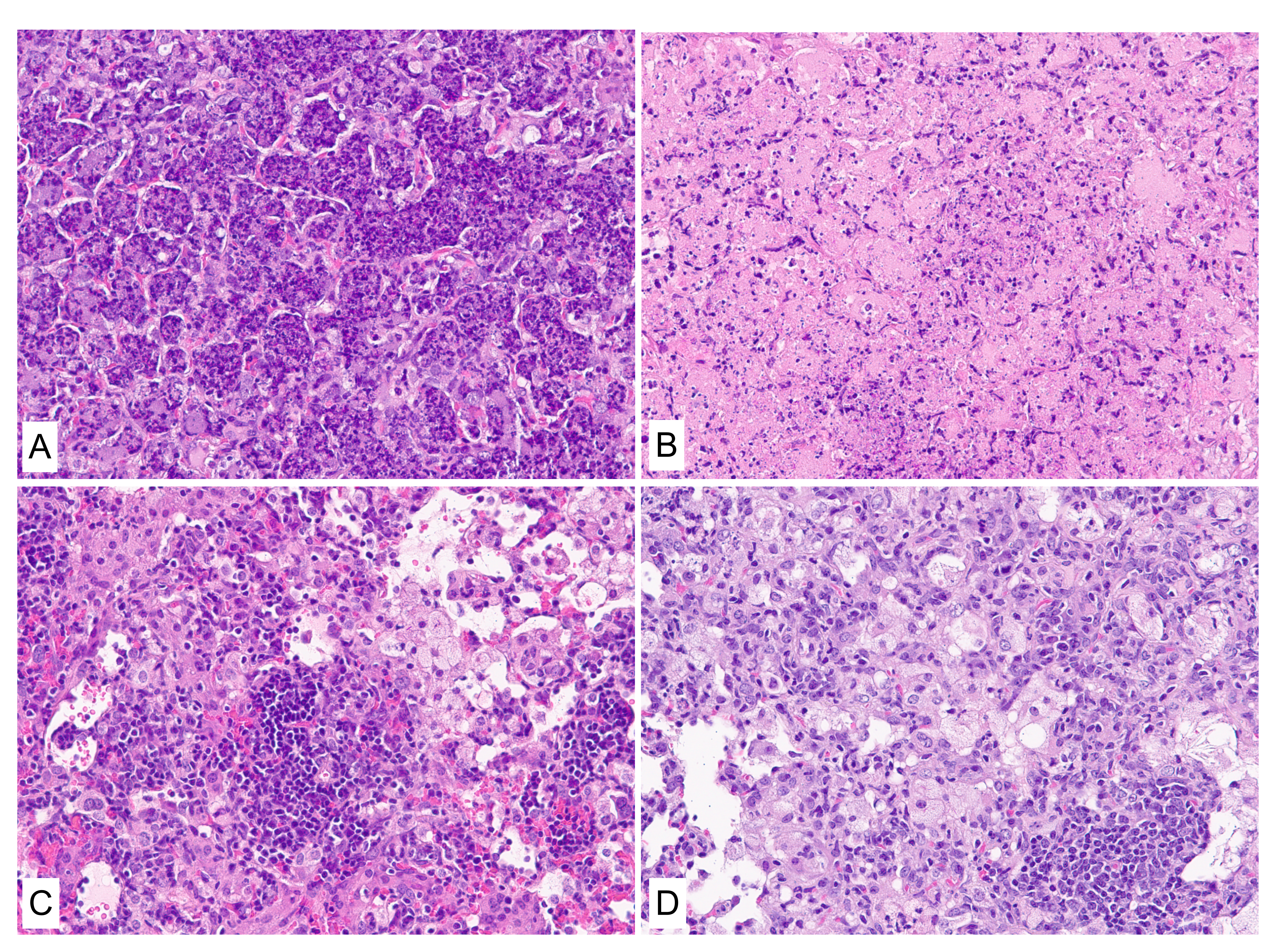

Supplement: S3 Fig — Lung lobes were formalin-fixed, paraffin-embedded, sectioned, and stained with carbol fuschin and counterstained with hematoxylin & eosin. Panels A and B: High magnification images of necrotizing lung lesions. One example contains abundant pyknotic nuclear debris (A) and one example contains abundant fibrin, eosinophilic cellular debris, and less nuclear debris (B). Panels C and D: High magnification images of non-necrotizing lung lesions. Both examples contain mostly viable cells, including macrophages, foamy macrophages, and foci of lymphocytes (400X). (TIF) [file ppat.1011915.s003.tif]

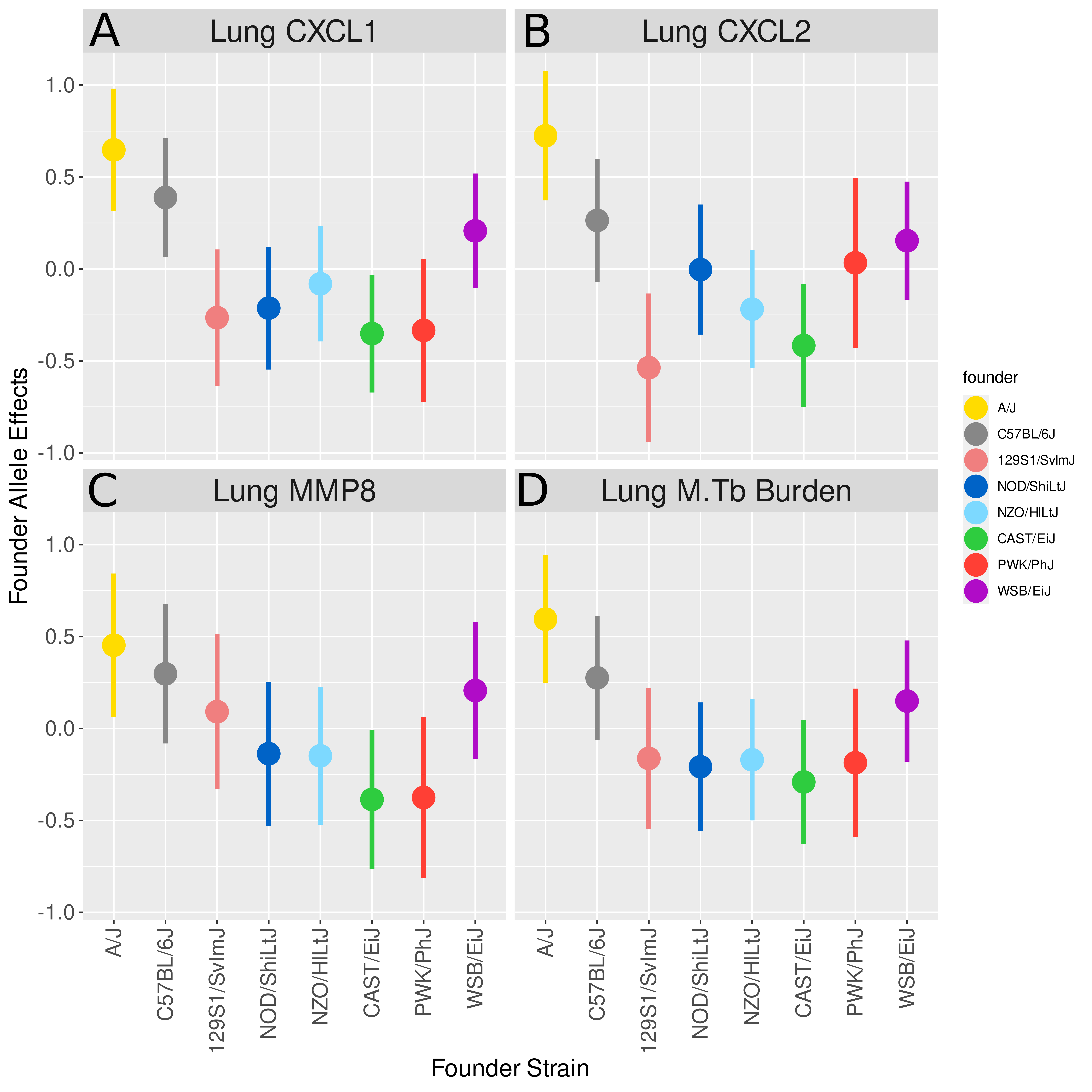

Supplement: S4 Fig — The pattern of founder allele effects of four correlated traits all with LOD > 6.0 on chromosome 1 at 155.36 Mb is similar. Panel A: Founder allele effects for CXCL1. Panel B: Founder allele effects for CXCL2. Panel C: Founder allele effects for MMP8. Panel D: Founder allele effects for M. tuberculosis burden. Founder strain names are on the horizontal axis and the standardized allele effect are on the vertical axis. Allele effects from QTL mapping results from n = 853 M. tuberculosis infected Diversity Outbred mice up to 250 days post infection. (TIF) [file ppat.1011915.s004.tif]

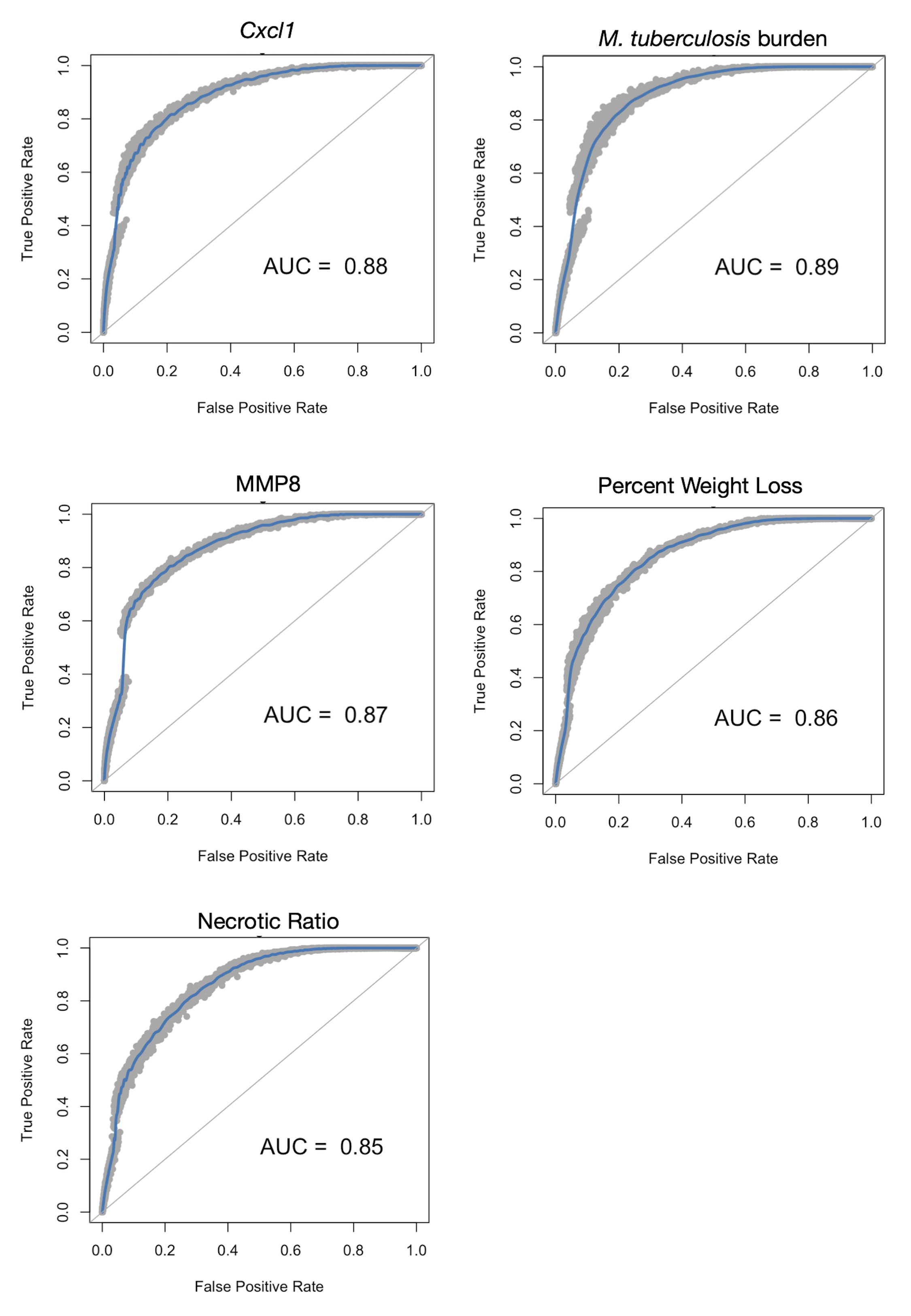

Supplement: S5 Fig — Each panel shows the true positive rate of the trained SVM as a function of the false positive rate for each trait. The area under the curve (AUC) is noted for each panel. (TIF) [file ppat.1011915.s005.tif]
