# Supplementary material for: Systems genetics uncover new loci containing functional gene candidates in Mycobacterium tuberculosis-infected Diversity Outbred mice
Source: PLoS Pathog. 2024 Jun 11;20(6):e1011915. doi: 10.1371/journal.ppat.1011915 (PMC11195971; doi:10.1371/journal.ppat.1011915)
Supplement: Attachment — Submitted filename: Gatti et al Response to Review.pdf [file ppat.1011915.s010.pdf]

Reviewer #1: Minor Comments

1. Comment References provided in at least two pieces of the text (line 46, Ref. to 8 works, and line 77, Ref. to 25(!) works in a row) look vague. The authors should make citations more specific.
    a. *Response We agree. We reorganized the references, so they are more specific and better placed. The number of citations was not reduced though.*

2. Comment Table 1: The Chr. 3 QTL is not new. Position of the locus containing a few S100A genes on the Chr. 3 coincides precisely with the TB-related QTL mapped in the work by Sanchez et al., Infect. Immun., 2003, doi: 10.1128/IAI.71.1.126-131.2003.
    a. *Response Thank you for the helpful reference. We reviewed and compared the positions. Sanchez et al in 2003 identified a QTL designated tbs1 (tuberculosis severity 1) on distal Chr. 3 associated with the positional marker D3Mit215 (115924105-115924250 bp) by crossing A/Sn and I/St inbred mouse strains. Based on position, we agree it is the same location as the DO QTL we identified using Diversity Outbred mice (peak 90.69 and interval 90.52-92.02 Mb). The Table and related sections of the manuscript have been revised accordingly, and reference cited. Thank you for the insight.*

3. Comment Figure 3A: It is difficult to see P values – overlapping with whiskers.
    a. *Response Figure 3A has been revised so the p-values overlap less with the whiskers.*

4. Comment Line 999 – Chr. 1 QTL, is this correct?
    a. *Response We cannot find Line 999, but the revision has been proofread and edited multiple times.*

Reviewer #2: Major and Minor Comments

1. Comment Legend for Figure 1 does not include a description of part B, please add details.
    a. *Response The description of panel B is now included.*

2. Comment Figure 4 is hard to interpret with the color scale. Please add Manhattan plots.
    a. *Response The important colors in Figure 4 are orange to yellow, corresponding to high LOD scores on each chromosome. Grey and black are purposefully dark shades because the LOD scores are not significant. We considered Manhattan plots, and tried them, but prefer visualization of the peaks by the heatmap as the correlated and colocalizing traits are apparent. We hope this is acceptable as author's preferred method of visualization.*

3. Comment Did early mortality correspond with mice from the first 2 DO cohorts that received the higher dose of *M. tuberculosis*?
    a. *Response No. A dose of about 100 M. tuberculosis colony forming units increases the percentage of the population with early mortality, but it is not the cause of all early mortality. Shown in:*
        i. *Niazi et al. Dis Model Mech. 2015 Sep;8(9):1141-53.*
        ii. *Kurtz et al. mSphere. 2020 Apr 15;5(2):e00097-20.*

4. Comment Line 318/Figure 2 – Higher magnification images should also be included so distinct pathological features can be seen.
    a. *Response The revised manuscript includes high magnification images in Supplementary Figure 3, also shown in:*
        i. *Niazi et al. Dis Model Mech. 2015 Sep;8(9):1141-53.*
        ii. *Kurtz et al. mSphere. 2020 Apr 15;5(2):e00097-20.*
        iii. Tavolara et al. EBioMedicine. 2020 doi: 10.1016/j.ebiom.2020.103094.

5. Comment There is some confusion about *Dots4*, in Table 1 it refers to a locus on Chr4 that affects S100A8 protein levels, however later in the text at Line 420 it refers to the multitrait locus on Chr17.
    a. *Response This was a typo and has been corrected. Additionally, all QTL names were revised so they match the guidelines in Standardized Genetic Nomenclature for Mice.*

6. Comment The authors should discuss what impact the missense or other SNPs in their candidate genes are predicted to have on protein structure or function. Are they in key functional domains? Important for post-translational modifications? In the discussion section, paragraphs starting with we identified X as a candidate gene should include the phenotype/locus for easy reference.
    a. *Response Table 2 now includes the predictions on protein functions based on the SNP location, alternate amino acid physiochemical properties, and score/qualitative provided from the public bioinformatics databases and tools in Ensembl, VEP and, SIFT. We revised the discussion section as suggested.*

7. Comment How were the 98 samples selected for expression analysis? Did they represent extreme phenotypes or a random selection of animals?
    a. *Response The samples were selected to include the range of phenotypes from infected mice with and without early mortality, including the range of M. tuberculosis lung burdens, and histopathological lesions.*

8. Comment Include tables with complete candidate gene lists for each QTL described.
    a. Response *We have added Supplemental File 1, which is an Excel workbook containing worksheets with protein-coding genes in each QTL.*

9. Comment Similarly, the genes with the top 10 highest functional scores should be ranked for QTLs where the authors used expression data and the trained SVM model to rank gene candidates.
    a. *Response We have added a Supplemental File 2, also an Excel fie, that identifies the top 10 functional candidates for each trait in each QTL.*

10. Comment The authors need to validate one of their candidate genes to warrant publication in a journal at the level of PLoS Genetics. The discussion mentions existing Zbtb20, Ier3, Vegfa knockout mice and although genetically deficient mice are not a perfect model for natural genetic polymorphisms, they may still inform an understanding of a given gene's role in disease.

a. _Response_ *We performed three different types of validation of DOQTL results, shown in revised Supplementary Figure 5. This included survival analysis of Diversity Outbred mice carrying PHK/PhJ alleles chr 17 MCH locus; quantifying S100A8 protein levels in lungs of M. tuberculosis infected PWK/PhJ and CAST/EiJ inbred founder strains; and in vivo infection of gene deficient mice. We selected the candidate with the highest LOD score (S100a8), obtained knockout mice from Dr Philippe Tessier at Laval University, and infected knockouts, heterozygotes, and wild-type C57BL/6J mice. Briefly, the absence of S100a8 gene had minimal impact on short-term survival, body weights, M. tuberculosis lung burden, or granuloma histopathological features when deleted from the on the C57BL/6 genetic background.*

Reviewer #3: Major and minor comments.

1. _Comment_ I would suggest to the authors that they create a flow chart outlining all the steps in the QC and integration process. It would be helpful to see the whole workflow/pipeline (i.e., where the probe/gene selection was performed as well as the normalization, correlation, QTL, etc.)
    a. _Response_ *This is a great idea. We have added a workflow diagram, Figure 5, to clarify the procedure for ranking positional candidate genes by functional score.*

2. _Comment_ It's unclear to me why they chose a SVM…It seems that if you pulled on the microarray data from the lung TB and plotted the first two principal components you could likely separate the majority of the variation in the data set which I would presume would be a result of genetics, TB disease severity and time (and possibly other covariates).
    a. _Response_ *We apologize for a lack of clarity. The SVM model is part of the network analysis that is downstream of the gene expression analysis. Briefly, the SVM uses gene-gene interaction weights to rank all genes in the genome according to functional association with a user-supplied gene set. In this case, the input gene list was the set of genes correlating with our mapped traits and the network analysis was applied to identify other genes that are functionally associated with these DEGs, and by extension the traits. The output ranking gives a natural way to prioritize among positional candidates, without a stringent requirement that candidates must be differentially expressed to be nominated, which would not be expected for example in the case where the causal variant influences protein coding. The choice of SVM for this analysis was motivated by extensive use of SVMs in this context by our group and others. We have added language to the section "Trait-related Gene Sets" to clarify the above.*

3. _Comment_ Under the section "support vector machine classifier training" The authors used the network from the following data set: http://fntm.princeton.edu and identified genes that had associations with the same functional groups. They determined these groups using gene ontology and KEGG pathways. An issue with this is Gene Ontology can have very vague classifications (biological process) and this could be drawing weak connections between gene pairs. This is a curated data set focusing on mouse and lung data and it's assumed that these pathways are

unique, but it would benefit readers to know (in the supplemental) which pathways were used in the classification of the SVM.

   a. Response *We agree with the reviewer that GO annotations can be vague. However, the FNTM gene networks that we used in this study were not developed by us, but rather are a public resource for precisely the kinds of gene prioritization problems we have here. We refer to the primary publication of this resource for an extensive discussion for how the authors curated GO terms to define ground-truth functional similarities and how they calibrated the final gene network weights. Our only input to the network analysis was the set of genes from our gene expression analysis. To bolster our case that these models learn important biology and provide sound gene rankings for candidate gene prioritization, we have included the machine learning outputs, specifically the receiver operator characteristic (ROC) curves (Supplemental Figure 5), which show that these models perform significantly better than chance at predicting correlated genes. We note, however, that these models are not expected to have perfect performance, and that many high ranking genes from outside the input set are expected to be trait relevant.*

4. Comment Also unclear what are the genetic backgrounds of these mice in the FNTM data set? Is most of the data B6 mice?
   a. Response *In the FNTM publication they delineate all data sets used to train their network models, which included a large fraction of the mouse gene expression data on the Gene Expression Omnibus at the time of the study. While we do not have a complete answer to the question as asked, we expect that the data sets used to train the FNTM models were dominated by mice with a B6 background. We certainly agree that were the networks trained with gene expression from a greater diversity of backgrounds, the networks and our subsequent inferences would likely be better. However, this is beyond the scope of the present study.*

5. Comment Under Trait-related Gene Sets can you clarify how many genes were identified within the QTLs? There were 500 genes calculated by their correlative values but were all these genes within the QTL region or were they just correlated by expression values? You list the QTL and key immune or inflammatory genes, but it would help to see (in the supplemental) a complete list of the genes identified. *We have included Supplemental Table 1, which contains the protein-coding genes which lie within each QTL interval.*
   a. Response *We have clarified this point in the section "Trait-related gene sets". The number of genes in each QTL, naturally, varied as a function of gene density and confidence interval size. We inconsistently mentioned the number of protein-coding positional candidate genes in the description of each* Dots *QTL, but now each description identifies this number. The choice of 500 genes was from our gene expression correlation analysis to the traits. These 500 genes were our input set to the downstream network analysis. The specific choice of 500 genes was made because of prior work showing that 500 input genes is an optimal number for these network analysis (*PMID: 21085640)

6. Comment In the SVM genes are ranked by their correlative value to train (or no-trait). In the downstream analysis why did you choose to rank genes functionally? I think this is acceptable,

but I think there are more statistically robust methods like differential expression. This also allows one to determine levels of statistical significance within the identified genomic loci with multiple test corrections and not across the whole genome.

   a. *Response* We hope the answers above clarified this confusion. Gene-trait correlations were assessed prior to SVM analysis. The effect of a gene on a trait can be mediated through mechanisms unrelated to transcript abundance, such as protein-coding variation, or translational efficiency. Thus, the goal of SVM analysis was to provide a functional ranking that would identify trait-relevant genes irrespective of differential expression.

7. Comment All Diversity Outbred mice and inbred founder strains were female. Could some of these results be gender specific?
   a. *Response Possibly but likely gender has less of an impact than genetically controlled immune responses. We observed that male DO mice show similar M. tuberculosis-induced phenotypes in Kurtz et al. mSphere. 2020 Apr 15;5(2):e00097-20. We had no QTLs on the X chromosome, and no candidate genes with roles in gender-specific differentiation, hormonal regulation, gonadal development, or reproductive pathways.*

8. Comment Is there a way to verify these targets from reported GWAS human studies? Have any of the identified genes been previously reported?
   a. *Response Great idea. We searched literature and databases and identified one candidate gene (mouse gene Ncf2) that was previously identified in human studies (human gene NCF2). In humans, a particular SNP in this gene was associated with reduced risk of pulmonary tuberculosis. This information and citation are included in the discussion.*
