# Supplementary material for: Systems genetics uncover new loci containing functional gene candidates in Mycobacterium tuberculosis-infected Diversity Outbred mice
Source: PLoS Pathog. 2024 Jun 11;20(6):e1011915. doi: 10.1371/journal.ppat.1011915 (PMC11195971; doi:10.1371/journal.ppat.1011915)
Supplement: Attachment — Submitted filename: Gatti et al PPATH Response.docx [file ppat.1011915.s011.docx]

31 March 2024

To:	PLoS Pathogens

Re:	Response to review of PPATHOGENS-D-23-02240 - [EMID:7e6fc9ec4b287c9c]

Dear Reviewers,

We are grateful for the careful review and positive comments. We addressed critiques and marked the revisions by blue font. Point-by-point responses with additional explanations are below:

**Major Issues**

**Reviewer 1**

Reviewer 1 stated that “Editors are aware that I have served as a reviewer during the first submission of the manuscript to PLoS Genetics, expressed my concerns and received adequate responses from the authors. Since the manuscript underwent notable additional improvements, I have no major concerns.”

**Reviewer 2**

None

**Reviewer 3**

*Critique* **(i)** The timelines and samples are difficult to follow and would be improved by including a timeline. **(ii)** Overall, it seems strange that progressor and controllers were not analyzed separately.

*Response*

(i) We clarified sampling and the sample size in the body of text, figures, and figure legends. When experimentation started, there were many unknowns: the range of phenotypes; correlates of pulmonary TB that could become quantified traits for genetic mapping; and sample sizes required to achieve statistical power. Therefore, the experiments were not driven by a single time point as we would not have a rational basis to select one time point. Instead, experimentation was performed to include the widest range of disease states, ensure the entire range of phenotypes could be sampled. Future studies can use the phenotypes herein to guide more refined – timepoint driven – trait quantification and genetic mapping.
(ii) Studies characterizing Progressors and Controllers have been analyzed separately by us and others, (in particular by Dr. Shabaana Khader’s group) for biomarker discovery, granuloma features, automated image analysis, predictive modeling, and direct comparison to humans. For analyses by class from our studies, we kindly refer the reviewer to:

- Koyuncu *et al*. PLoS Pathog. 2021 Aug 17;17(8):e1009773.
- Tavolara *et al* EBioMedicine. 2020 Dec;62:103094.
- Tavolara *et al*. EBioMedicine. 2021 May;67:103388.
- Niazi *et al*. Dis Model Mech. 2015 Sep;8(9):1141-53.
- Additionally, we have a revised manuscript in review for Infection & Immunity.

*Critique* Supplemental Fig. 3A is listed as pyknotic nuclear debris in a necrotizing granuloma. The authors should **(i)** clarify how this was confirmed vs alveolar accumulation of mostly intact PMNs? **(ii)** Does accumulation alone meet criteria as necrotizing when the alveolar wall is intact? There are several interesting granuloma features, as shown in Fig. 2 (e.g., Fig. 2E) that are mentioned in text but not illustrated. Since these lesions are similarly scored as necrotizing when developing the relationships, **(iii)** this distinction requires clarification.

*Response*

(i) The last author (GB) is a board certified veterinary pathologist and can confirm by specialty training and experience that Supplemental Figure 3A is pyknotic nuclear debris in alveolar airspaces by the following inclusion and exclusion morphological criteria. Pyknotic nuclear debris from any cell source is recognized by the small condensed, variably sized hyperchromatic fragments in the absence of cells with intact cytoplasmic borders. The morphology shown is not consistent with intact neutrophils. Intact neutrophils have intact nuclei that are larger than the pyknotic debris present, and intact neutrophils have lobulated or segmented nuclei, variable nucleoli, with cytoplasm visible as a semi-clear space surrounding the intact nucleus or with a visible cytoplasmic membrane. There is agreement with the reviewer’s conclusion that the cellular origin of the pyknotic debris is most consistent with dead and dying neutrophils; however, dead and dying monocytes/macrophages and/or dead and dying myeloid-derived suppressor cells cannot be ruled out. Definitive determination of origin of the debris is not feasible using morphology alone, although an abundance of neutrophils with neutrophils as the main cell origin is consistent with gene expression profiling and analyses in Koyuncu et al. PLoS Pathog. 2021 Aug 17;17(8):e1009773.
(ii) There are certainly use-case preferences amongst pathologists who may agree or disagree with the term necrotizing. In context here, necrotizing characterizes the inflammatory response – that the inflammatory cells *themselves* are undergoing necrosis. The term does not have to include concurrent necrosis of tissue parenchyma (e.g., alveolar septae) as that can occur as a separate disease process and may have different initiating mechanisms although both lesion types are present in most of the necrotic granulomas in Diversity Outbred mice.
(iii) We agree there are many interesting granuloma features, but it is beyond the scope of this work to compile and describe all of them as most were not quantified for genetic mapping and only granuloma necrosis was quantified. One point to clarify, lesions were not manually scored because it was not feasible to manually and reproducibly score thousands of lung tissue sections with sufficient dynamic range for QTL mapping. Instead, to quantify granuloma necrosis, the area of necrotic regions was calculated using a validated, deep learning algorithm that we specifically developed for this purpose. The deep learning algorithm and pathologist validation is described in Tavolara et al EBioMedicine. 2020 Dec;62:103094.

*Critique* Supplemental Figure 6 is confusing. **(i)** Survival data for mice with at least one PWK/PhJ allele indicates a survival disadvantage. **(ii)** Comparisons to the CAST/EiJ strain (low allelic expression) indicate Mtb lung burden is no different, yet only Mtb lung burden is shown for the S100A8 KO mice. **(iii)** It is unclear why histology, cytokines, or survival was not evaluated for S100A8 KO mice, given that these outcomes are important and key inputs to the model. If specimens are available, these analyses should be included.

*Response*

(i) We apologize and have revised the text and the figure. The panels in Supplemental Figure 6 demonstrate validity of method and show how results from genetic mapping may be used. Because genetic mapping identified *pwk* with highest allele effects at the Chromosome 17 QTL (Figure 9), we were curious whether *pwk* alleles at the same locus impacted survival and to what degree. To evaluate this, we plotted and analyzed survival of *M. tuberculosis*-infected Diversity Outbred mice with one or more *pwk* alleles *vs* other alleles at the H-2 locus, which is a major immune response region on Chr 17. The results in Panel A show that Diversity Outbred mice with *pwk* alleles at the H-2 locus had reduced survival compared to other alleles.
(ii) Because the locus on chromosome 3 had the highest LOD score, narrowest interval, distinct founder allele effects, and the gene candidate was the *S100a8* gene itself, the locus was selected for additional study. With *cast* and *pwk* alleles at the Chromosome 3 locus having opposite effects, we hypothesized that PWK/PhJ inbred mice would have higher levels of S100A8 than CAST/EiJ inbred mice after *M. tuberculosis* infection, and this was shown to be true (Panel B). We removed the CFU results comparing these two founder strains.
(iii) For the same reason as (ii), we obtained, bred, and infected S100A8 KO, heterozygous, and WT C57BL/6 mice. Following low dose aerosol infection, we observed no significant differences in *M. tuberculosis* burden at 3 early timepoints post infection (Panel C). We performed histology, which is now shown at the 50 day timepoint (Panels D, E, F); and monitored body weights and survival out to approximately 100 days. There were no substantive differences in any of the readouts. We do (did) not report or show all results because “data not shown” was disallowed in the submission process. We have considered adding additional Supplemental figures; however, we decided to refrain from adding more supplemental figures because the body of work is large already and more results from S100A8 KO C57BL/6 mice do not fundamentally change the conclusions drawn from nearly one thousand Diversity Outbred mice that were studied.

**Minor Issues -  Editorial and Data Presentation Modifications**

**Reviewer 1**

“My minor concerns expressed previously were also met.”

**Reviewer 2**

*Critique* Table 2 would be strengthened if it displayed a column with brief descriptions of the known gene functions…Table 2 does not describe gene function or SNP effects.

*Response* We apologize for the inconvenience. This is a great idea, and we have revised Table 2 as suggested with a column “known protein functions”. The reviewer should now see the following headers:

| **QTL** | **Candidate** | **Known protein functions** | **MVAR Annotations*** | **Alleles with SNPs**** | **Predicted effects of missense SNPs on proteins.** |
| --- | --- | --- | --- | --- | --- |

*Critique* The authors report that the s100a8 heterozygote (+/-) and knockout (-/-) mutations in C57BL/6 mice showed minimal impact on ability to control Mtb lung CFUs. This contrasts with Scott et al. JCI 2020 (cited as ref. 91) who found that s100a8/a9 deficiency was significantly protective with lower lung CFU counts. It would be helpful for the authors to cite Scott et al. in this section of the text and to comment on the discordant results either in the Results or in the Discussion.

*Response* Thank you, this is also a great idea. We have updated the discussion to put our results in better context with Scott *et al.*, attempting to capture the similarities, differences, and uncertainties. The two proteins (S100A8 and S100A9) do have independent and inter-dependent biological functions, depending on form (monomer, dimer, heterodimer, multimer); which receptors are engaged (TLR4, RAGE); and context/genetic background. *M. tuberculosis* CFU from our work using S100A8 KO C57BL/6 aligns CFU from S100A9 KO C57BL/6 but it is difficult to be certain because the genes knocked out were different, we did not examine long term infection or survival while Scott *et al* did.

*Critique* Line 480 refers to Fig. 9E, but I cannot find such a figure.

*Response* This error has been corrected to 9D.

**Reviewer 3**

*Critique* **(i)** How many animals from each group are represented in data sets is also difficult to assess. Figure legends do not consistently indicate this information. **(ii)** Also, is the data from all groups (progressors, etc.) pooled for QTL analysis? If so, how were comparisons among these groups made to inform the pooling criteria? **(ii)** Suppl Fig. 1 indicates 195 progressor and 145 controller mice. **(iv)** Gene expression is from 98 mice, but how were they selected? **(v)** What was the basis of selection of analytes for the ELISA panel re: discovery? The panel is highly customized.

*Response*

(i) We clarified sampling and the sample size in methods, body of text, figures, and figure legends.
(ii) Quantitative trait locus mapping requires aggregate data to minimize bottlenecks that could produce biased results or spurious associations due to absent genotypic and phenotypic contributions from the missing groups. We did not make comparisons to select specific responders for inclusion in QTL mapping. Instead, we used all available data for multiple rounds of QTL mapping. The mapping results from mice up to 250 days post-infection is shown because that data set included a sample size that met power calculations, samples that passed QA/QC, and because it was the least noisy, produced the most numbers of significant loci. Our methods section explains the pooling and data normalization steps and the results section explains the justification for using the data sets from mice up to 250 days post infection.
(iii) Supplemental Fig 1 shows the weight changes over time only from mice where infection was allowed to progress along its entire natural course, and were confirmed morbidity was due to pulmonary TB. Infected mice that were euthanized prior to the development of morbidity are not included in Supplemental Fig 1 because their body weights changes over time would be incomplete - truncated early – and potentially give a false sense of the spectrum of disease progression.
(iv) The rationale for a subset of mice sampled for gene expression is included in the revised manuscript. Briefly, mice were carefully selected to represent the spectrum of observable phenotypes. Selection details be found in Koyuncu *et al*. PLoS Pathog. 2021 Aug 17;17(8):e1009773.
(v) The rationale for trait selection is included in the revised manuscript. Briefly, quantified traits were selected for biological relevance to span clinical, granuloma, microbiological, and inflammatory mediators that are known to positively correlate with disease, or were being investigated as biomarker candidates of pulmonary TB, or have been shown to negatively regulate immune/inflammatory responses in *M. tuberculosis*. Briefly,
    a. Clinical correlate of disease was weight loss.
    b. Granuloma correlate of disease was necrosis.
    c. Microbiological correlate of disease was *M. tuberculosis* CFU.
    d. Chemokine correlates of disease were CXCL1, CXCL2, CXCL5.
    e. The proinflammatory cytokine correlate of disease was TNF.
    f. Biomarker candidates were S100A8, MMP8, VEGF
    g. Immune regulatory cytokine was IL-10.

*Critique* Figure 5 overview is not a standalone figure, should be included within a figure as a visual, or moved to supplemental data.

*Response* We kindly request to keep Figure 5 as a standalone as previous reviewers requested it.

*Critique* Fig. 3. P values are difficult to see, and numbers of replicates are not presented in the figure or figure legend. What timepoint post-infection is this? Are these Pearson correlation values shown significant? It is not clear if that is what the size of the elliptical shape that indicates strength of the association represents.

*Response* Figure 3 has been adjusted. The numbers of mice and time point are now included in the legend; and statistically significant correlations are shown by an asterisk. We have also emphasized in the legend that the color of the ellipses shows positive (blue) or negative (red) correlation, and the shape indicates the strength of correlation with narrow shape indicating higher correlation.

*Critique* Recommend use of greater and lesser as compared to higher and lower. Correct the following:

Line 351: "remainder (of) lung"

Line 420: needs “a” in “Dots4 is new QTL”

Line 443 Dots6 is new QTL

Line 540: Mice this temporarily?

Line 625: we multiply

Line 646 punctuation in Dr

*Response* We included greater and lesser. In some sentences “high” and “low” are retained for describing allele effects as these terms are typical for the field, and for describing magnification power for histopathological images. The typos were corrected, as well as a few others that we noticed when revising.

*Critique* Why only look at Mtb burden with the S100a8 KO mice and not necrosis and inflammation, outcomes used to develop linkages?

*Response* We obtained, bred, and infected S100A8 KO, heterozygous, and WT C57BL/6 mice. Following low dose aerosol infection, we observed no significant differences in *M. tuberculosis* burden at 3 early timepoints post infection (Panel C). We performed histology, which is now shown at the 50 day timepoint (Panels D, E, F); and monitored body weights and survival out to approximately 100 days. There were no substantive differences in any of the readouts. We do (did) not report or show all results because “data not shown” was disallowed in the submission process. We have considered adding additional Supplemental figures; however, we decided to refrain from adding more supplemental figures because the body of work is large already and additional results from S100A8 KO C57BL/6 mice do not change the conclusions drawn from nearly one thousand Diversity Outbred mice that were studied.

Sincerely,

Gillian Beamer
